# Integration of proteomic and transcriptomic profiles identifies a novel PDGF-MYC network in human smooth muscle cells

**DOI:** 10.1186/s12964-014-0044-z

**Published:** 2014-08-01

**Authors:** Wei Yang, Aruna Ramachandran, Sungyong You, HyoBin Jeong, Samantha Morley, Michelle D Mulone, Tanya Logvinenko, Jayoung Kim, Daehee Hwang, Michael R Freeman, Rosalyn M Adam

**Affiliations:** 1 Cancer Biology Program, Samuel Oschin Comprehensive Cancer Institute, Cedars-Sinai Medical Center, Los Angeles 90048, CA, USA; 2 Departments of Surgery and Biomedical Sciences, Cedars-Sinai Medical Center, Los Angeles 90048, CA, USA; 3 Urological Diseases Research Center, Boston Children’s Hospital, Boston 02115, MA, USA; 4 Department of Surgery, Harvard Medical School, Boston 02115, MA, USA; 5 School of Interdisciplinary Bioscience and Bioengineering, POSTECH, Pohang, Republic of Korea; 6 Clinical Research Center, Boston Children’s Hospital, Boston 02115, MA, USA; 7 Center for Systems Biology of Plant Senescence and Life History, Institute for Basic Science, DGIST, Daegu 711-873, Republic of Korea; 8 Urological Diseases Research Center, John F. Enders Research Laboratories, Boston Children’s Hospital, Rm 1061.1, Boston, 300 Longwood Avenue, Boston 02115, MA, USA

**Keywords:** Visceral smooth muscle, Proteomics, Transcriptomics, Network analysis, PDGF, DIAPH3, ProteomeXchange PXD000624

## Abstract

**Background:**

Platelet-derived growth factor-BB (PDGF-BB) has been implicated in the proliferation, migration and synthetic activities of smooth muscle cells that characterize physiologic and pathologic tissue remodeling in hollow organs. However, neither the molecular basis of PDGFR-regulated signaling webs, nor the extent to which specific components within these networks could be exploited for therapeutic benefit has been fully elucidated.

**Results:**

Expression profiling and quantitative proteomics analysis of PDGF-treated primary human bladder smooth muscle cells identified 1,695 genes and 241 proteins as differentially expressed versus non-treated cells. Analysis of gene expression data revealed MYC, JUN, EGR1, MYB, RUNX1, as the transcription factors most significantly networked with up-regulated genes. Forty targets were significantly altered at both the mRNA and protein levels. Proliferation, migration and angiogenesis were the biological processes most significantly associated with this signature, and MYC was the most highly networked master regulator. Alterations in master regulators and gene targets were validated in PDGF-stimulated smooth muscle cells in vitro and in a model of bladder injury in vivo. Pharmacologic inhibition of MYC and JUN confirmed their role in SMC proliferation and migration. Network analysis identified the diaphanous-related formin 3 as a novel PDGF target regulated by MYC and JUN, which was necessary for PDGF-stimulated lamellipodium formation.

**Conclusions:**

These findings provide the first systems-level analysis of the PDGF-regulated transcriptome and proteome in normal smooth muscle cells. The analyses revealed an extensive cohort of PDGF-dependent biological processes and connected key transcriptional effectors to their regulation, significantly expanding current knowledge of PDGF-stimulated signaling cascades. These observations also implicate MYC as a novel target for pharmacological intervention in fibroproliferative expansion of smooth muscle, and potentially in cancers in which PDGFR-dependent signaling or MYC activation promote tumor progression.

## Introduction

Smooth muscle-rich hollow organs such as the vasculature, airways, gut and urinary tract undergo tissue remodeling following injury. These alterations in tissue structure include cellular hypertrophy and hyperplasia, increased synthesis and secretion of extracellular matrix, dedifferentiation of smooth muscle cells (SMC) and progressive loss of normal contractile function. Importantly, even after removal or attenuation of the inciting stimulus, tissue damage resulting from pathologic remodeling persists, sometimes indefinitely, and there are typically limited options for treatment.

Among the soluble factors implicated in the pathologic responses of SMC to injury, the potent mitogen and motogen platelet-derived growth factor-BB (PDGF-BB) has emerged as an important soluble driver [1]. PDGF-BB elicits biological effects, such as proliferation and migration, through dimerization and activation of PDGF receptor (PDGFR) tyrosine kinases and initiation of downstream kinase cascades that impinge on transcriptional complexes (reviewed in [2]). Signaling through the PDGFR axis has been implicated in a range of pathological conditions, including atherosclerosis, airway remodeling in asthma [3],[4] and fibroproliferative changes in the bladder wall [5]. However, neither the molecular basis of the PDGFR signaling repertoire, nor the extent to which specific elements within these cascades could be exploited for therapeutic benefit has been fully elucidated.

The downstream targets of PDGFR activation in smooth muscle have, for the most part, been defined at the level of small numbers of proteins or genes [5]–[8]. Expression profiling of smooth muscle exposed to PDGF has thus far been restricted to SMC of vascular origin, and has identified NFAT family members and target genes as important effectors of vascular SMC behavior in the setting of vascular injury [9],[10]. Genome-wide evaluation of PDGF-stimulated visceral smooth muscle gene expression has yet to be reported. Several groups, including our own, have employed mass spectrometry-based proteomics to interrogate PDGF-induced changes in cells of mesenchymal origin [11]–[15]. In a previous study, we used isotope-coded affinity tagging (ICAT) analysis coupled with mass spectrometry to quantify PDGF-induced protein alterations in a human visceral SMC sub-proteome [14]. In that study we observed marked enrichment in proteins associated with endocytosis and the cytoskeleton in lipid raft microdomains of cells treated with PDGF, consistent with other studies linking PDGF to alterations in cell morphology and the actin cytoskeleton.

In this study, we present the first integrated analysis of gene expression and proteome-level alterations in human visceral SMC challenged with PDGF.

## Results

### Gene expression regulated by PDGF

In order to interrogate global responses to PDGF-BB at both gene and protein levels, we used primary human bladder smooth muscle cells (pBSMC) to perform RNA expression profiling in concert with quantitative analysis of the entire proteome using the SILAC method. Expression of PDGFRα and PDGFRβ isoforms was verified in pBSMC by real-time RT-PCR and immunoblot analysis (Figure S2A & S2B (see Additional file 1)). Cells subjected to triplex SILAC labeling were treated with 1 nM PDGF-BB for 0, 4 or 24 h. Total protein lysates were analyzed using mass spectrometry, and total RNA was analyzed by expression profiling (workflow shown in Figure S2, (see Additional file 2)).

**Figure 1:**
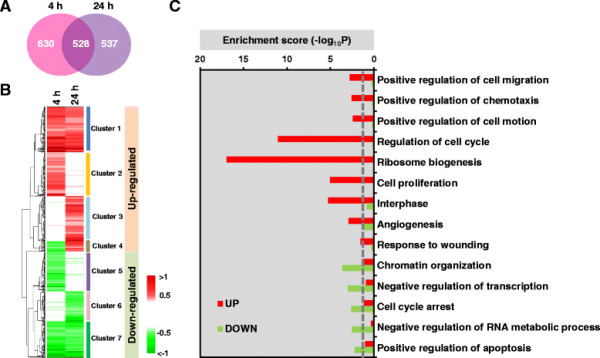
**Transcriptome analysis of pBSMC perturbed by PDGF-BB. (A)** Venn diagram depicts the proportion of DEGs in 4 h and 24 h microarray data sets. **(B)** Heatmap displaying differential expression patterns of 1,695 DEGs at 4 h and 24 h compared to 0 h. The DEGs were categorized into two groups: ‘Up-regulated’ (Cluster 1-4) and ‘Down-regulated’ (Cluster 5-7). The color shows increased (red) and decreased (green) expression. **(C)** Gene Ontology Biological Processes (GOBPs) enriched by DEGs. The bar graphs represent -log_10_(p), where *p* is the enrichment *p*-value from DAVID software.

Microarray data were assessed and determined to be of high quality (Figure S3 (see Additional file 3)); a high degree of reproducibility was observed based on inter- and intra-group variation of the arrays, with all pairwise correlation coefficients between samples >0.98. A total of 1695 differentially expressed genes (DEGs) with overall *p* <0.05 (Table S1 (see Additional file 4)) were identified at either 4 or 24 h using an integrative statistical method previously reported ([16], Materials and Methods). Of these, 528 DEGs were significantly changed at both 4 h and 24 h following PDGF treatment, while 630 and 537 DEGs were significantly changed only at the 4 or 24 h time point, respectively (Figure 1A). DEGs were grouped into clusters (Clusters 1 to 7), based on time-dependent differential expression patterns, by hierarchical cluster analysis. The seven clusters could be sub-categorized into those representing up-regulated genes (Clusters 1 to 4) and those reflecting down-regulated genes (Clusters 5 to 7). These data showed that 487 (88%) of the 528 DEGs identified at both times were consistently up- or down-regulated (Clusters 1 or 7 in Figure 1B), while 63 (12%) of the 528 genes perturbed at both times were down-regulated at 4 h but up-regulated at 24 h (Cluster 4 in Figure 1B). Functional enrichment analysis of Gene Ontology Biological Processes using Database for Annotation, Visualization and Integrated Discovery (DAVID) software suggested that cell cycle transit, cell proliferation, cell migration and motility, ribosome biogenesis and angiogenesis were the most prominent biological processes in the group of genes up-regulated by PDGF, whereas cell cycle arrest, chromatin organization and apoptotic pathways were the most prominent processes in the down-regulated group (Figure 1C).

**Figure 2:**
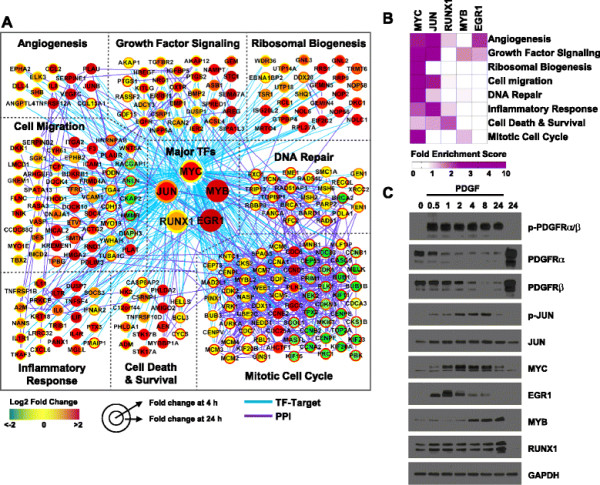
**A network model describing major cellular pathways regulated by five key TFs in response to PDGF-BB stimulation. (A)** The network was reconstructed using 255 target genes that belong to the overrepresented pathways. The node and node border colors represent log2 scale fold changes of mRNA expression at 4h and 24 h, respectively: genes decreasing at 4 h or 24 h compared to 0 hour are represented in green while those which increased at 4 h or 24 h are represented in red. The blue and cyan lines in the network indicate protein-protein interaction and TF-target interaction, respectively. The network nodes were arranged into several modules according to KEGG pathway information and Gene Ontology biological processes of the corresponding genes. **(B)** Two-dimensional map showing fold enrichment scores (FES) representing the contribution of TFs to the individual modules. The color gradient of each rectangle indicates the magnitude of the FES in the corresponding module. **(C)** Immunoblot analysis of time-dependent changes in PDGFR phosphorylation and putative master regulators in response to PDGF-BB treatment for the indicated times. The blot is representative of at least 3 independent trials.

To identify key transcription factors (TFs) involved in these gene expression alterations, we collected TF-target interaction data from six databases (TRED [17], EEDB [18], mSigDB [19], Amadeus [20], bZIPDB [21], and OregAnno [22]) and then identified TFs having significant numbers of DEGs as their targets (Materials and methods). Significantly up-regulated DEGs were mainly downstream targets of EGR1, JUN, MYB, RUNX1, and MYC (Figure 2A) while the significantly down-regulated DEGs were largely regulated by DDIT3, NFAT5, and SOX5 (data not shown). The up-regulated DEGs were enriched in eight biological processes: angiogenesis, growth factor signaling, ribosomal biogenesis, cell migration, inflammatory response, cell death and survival, mitotic cell cycle, and DNA repair (Figure 2B). In addition, the enrichment analysis showed that MYC targets were significantly enriched in all 8 processes and JUN targets were enriched in 6 out of the 8 processes, indicating that MYC and JUN are the two most prominent TFs downstream of PDGF in pBSMCs. Consistent with these results, a time-dependent assessment of these TFs confirmed that expression and/or phosphorylation of EGR1, JUN, MYB, RUNX1, and MYC was increased (Figure 2C) while that of DDIT3, NFAT5, and SOX5 was decreased by PDGF treatment at some but not all time points within 24 h (Figure S1D (see Additional file 1)).

### Protein expression regulated by PDGF

**Figure 3:**
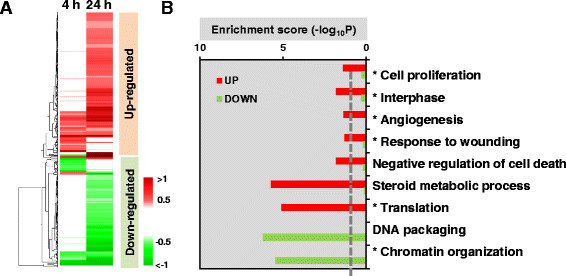
**Proteome analysis of pBSMC in response to PDGF-BB treatment. (A)** Heatmap displaying differential expression patterns of 241 DEPs at 4 h and 24 h compared to 0 h. The color shows increased (red) and decreased (green) expression. **(B)** Major cellular processes enriched by DEPs. Terms with an asterisk represent cellular processes common to both DEPs and DEGs. Functional enrichment analysis of up- and down-regulated proteins was performed using DAVID software. The bar graphs represent -log_10_(p), where *p* is the enrichment *p*-value from DAVID software.

To identify proteins regulated by PDGF, triplex SILAC analysis was performed in three replicates. A total of 2489 proteins were identified with FDR < 0.01. Representative mass spectra of SILAC peptide triplets are shown in Figure S4 (see Additional file 5). After quality assessment, 241 differentially expressed proteins (DEPs) with overall *p* < 0.05 (Table S2 (see Additional file 6)) were identified using integrated statistics ([16], Materials and methods). Hierarchical clustering showed that the DEPs were broadly grouped into up- and down-regulated clusters, with the majority of DEPs only significantly differentially expressed at 24 h (Figure 3A). Enrichment analysis of Gene Ontology processes indicated that cell proliferation, response to wounding, angiogenesis, translation and steroid metabolic pathways were significantly up-regulated. Conversely, DNA compaction and chromatin organization pathways were down-regulated (Figure 3B). Biological processes common to the transcriptome and proteomic profiles are indicated by asterisks.

### Integration of microarray and SILAC datasets

**Figure 4:**
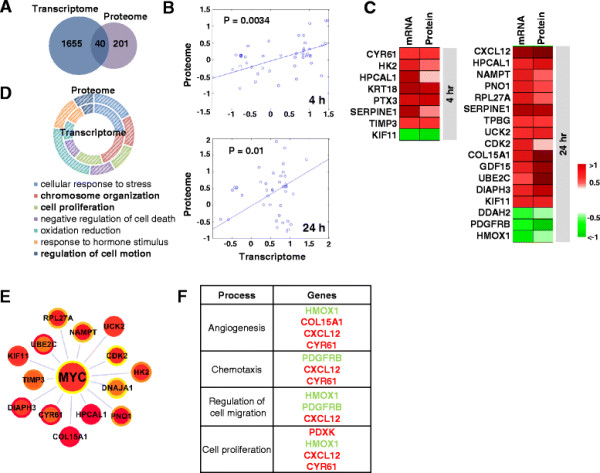
**Integration and comparison of DEGs and DEPs. (A)** Venn diagram depicts overlap between DEGs (n = 1695) and DEPs (n = 241), leading to 40 common gene and protein species. **(B)** Differential expression of these 40 molecules was significantly correlated (p ≤ 0.01, Spearman rank correlation test). **(C)** Heatmap shows the differential expression pattern of 22 common genes/proteins in response to 4 h and 24 h PDGF-BB treatment (HPCAL1, SERPINE1 and KIF11 are represented at both time points). **(D)** Enriched biological processes common to DEGs and DEPs. **(E)** MYC-centric network describing putative regulation of common gene/protein signature. The node and node border color represent expression changes of mRNA and protein, with the color showing increased (red) and decreased (green) expression. **(F)** The 22 common mRNA and protein species were classified functionally into angiogenesis chemotaxis, cell migration, or proliferation.

Next we performed an integrated analysis to explore the concordance between mRNA and protein levels in PDGF-treated pBSMCs. The correlation coefficient between mRNA and protein levels in pBSMCs treated without or with PDGF ranged from 0.41 to 0.45 (Figure S5 (see Additional file 7)). This is consistent with a previous global-scale correlation study showing that the coefficient of determination between mRNA and protein copy numbers in mouse NIH3T3 fibroblasts is 0.41 [23]. Among the 1695 DEGs and 241 DEPs, 40 targets were significantly changed at both mRNA and protein levels (Figure 4A, Table S3 (see Additional file 8)) and the changes at both levels were significantly correlated (p ≤ 0.01) (Figure 4B). 22 mRNA and protein species were consistently up- or down-regulated at 4 and 24 h (Figure 4C). Despite only 40 shared species, there was remarkable similarity in biological processes represented by the DEGs and DEPs (Figure 4D). This indicates that the shared alterations induced by PDGF are clearer at the cellular process or pathway levels than at the molecular level. Computational integration of all known transcription factors and their predicted potential to regulate the 40 shared RNA and protein species, identified MYC as the central transcriptional regulator of this signature (Figure 4E). The dominant biological processes represented by this signature were angiogenesis, chemotaxis, regulation of cell migration and cell proliferation (Figure 4F).

### Target validation in vitro and in vivo

**Figure 5:**
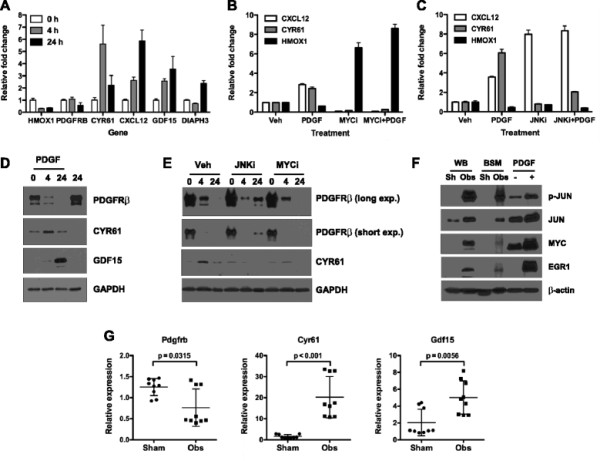
**Target validation in vitro and in vivo. (A)** Validation of mRNA levels of a subset of PDGF-regulated genes (HMOX1, PDGFRB, CYR61, CXCL12, GDF15 and DIAPH3) in pBSMC. **(B, C)** PDGF-induced changes in expression of the CXCL12, CYR61 and HMOX1 genes were evaluated in the context of MYC **(B)** and JNK/JUN **(C)** inhibition, using real time RT-PCR analysis. **(D)** Protein level changes in PDGFRβ, CYR61 and GDF15 in response to PDGF treatment for different times were verified by immunoblot analysis. Data are representative of three independent trials. **(E)** Sensitivity of PDGF-induced changes in PDGFRβ and CYR61 and to inhibition of JNK and MYC was assessed by immunoblot analysis. The long exposure is included to appreciate differences in sensitivity of PDGFRβ to JNK and MYC inhibition. **(F)** Immunoblot analysis of whole bladder tissue (WB, n = 2, pooled) or bladder smooth muscle (BSM, n = 5, pooled) from mice subjected to sham surgery (Sh) or outlet obstruction (Obs) were blotted with the indicated antibodies; serum-depleted pBSMC treated without (-) or with 1 nM PDGF-BB for 2 h (+) were included as negative and positive controls respectively. **(G)** Bladder smooth muscle tissues from sham-operated (Sham, n = 3) or obstructed mice (Obs, n = 3) were subjected to real-time RT-PCR analysis for the indicated transcripts.

The up- or down-regulation of a cohort of the molecules most significantly associated with the shared processes was validated by real-time RT-PCR analysis. As shown in Figure 5A, expression of HMOX1, PDGFRB, CYR61, CXCL12, GDF15 and DIAPH3 displayed time-dependent changes in expression following PDGF treatment. Findings presented in Figure 4 implicate MYC as a central regulator of the pBSMC response to PDGF. Notably, JUN/AP-1 also emerged from this global analysis (Figure 2), a finding that appears to confirm a series of published studies that identified JUN/AP-1 as a key regulator of mechanical signals in pBSMC [5],[24]–[28]. To probe the functional significance of these observations, we determined the impact of pharmacologic inhibition of MYC and JUN activation on expression of a subset of the validated gene targets. After confirming that MYC and JUN were effectively inhibited with the MYC inhibitor 10058-F4 (hereafter MYCi, [29]) and the JNK inhibitor SP600125 (hereafter JNKi) respectively, in pBSMCs (Figure S6 (see Additional file 9)), expression of 3 PDGF targets (HMOX1, CXCL12, and CYR61) was assessed by real-time RT-PCR. MYCi suppressed PDGF-regulated expression of all 3 targets, (Figure 5B) whereas JNKi only suppressed PDGF-regulated expression of HMOX1 but not of CXCL12 or CYR61 (Figure 5C). As independent validation of the network, additional targets were verified at the protein level (Figure 5D) and shown to be differentially sensitive to pharmacologic inhibition of JUN or MYC. PDGF-induced down-regulation of PDGFRβ was attenuated following inhibition of JNK, but insensitive to MYC inhibition. In contrast, inhibition of either JNK or MYC attenuated PDGF-stimulated up-regulation of CYR61 (Figure 5E).

To extend these findings, we determined whether signaling pathways and targets were altered in a mouse model of bladder injury. A previous study from our group demonstrated acute activation of the PDGFR axis and downstream effectors in response to bladder wall distension in rodents [5],[28]. As shown in Figure 5F, acute obstruction injury increased the level and/or phosphorylation of 3 transcription factors -- JUN, MYC, and EGR1 -- identified as key regulatory nodes in PDGF-stimulated transcription (Figure 2). In addition, expression of Pdgfrb, Cyr61 and Gdf15 transcripts was altered in the bladder injury model in a manner consistent with that observed following PDGF treatment of pBSMC (Figure 5G), further validating the network predictions.

### Functional interrogation of key regulatory nodes

**Figure 6:**
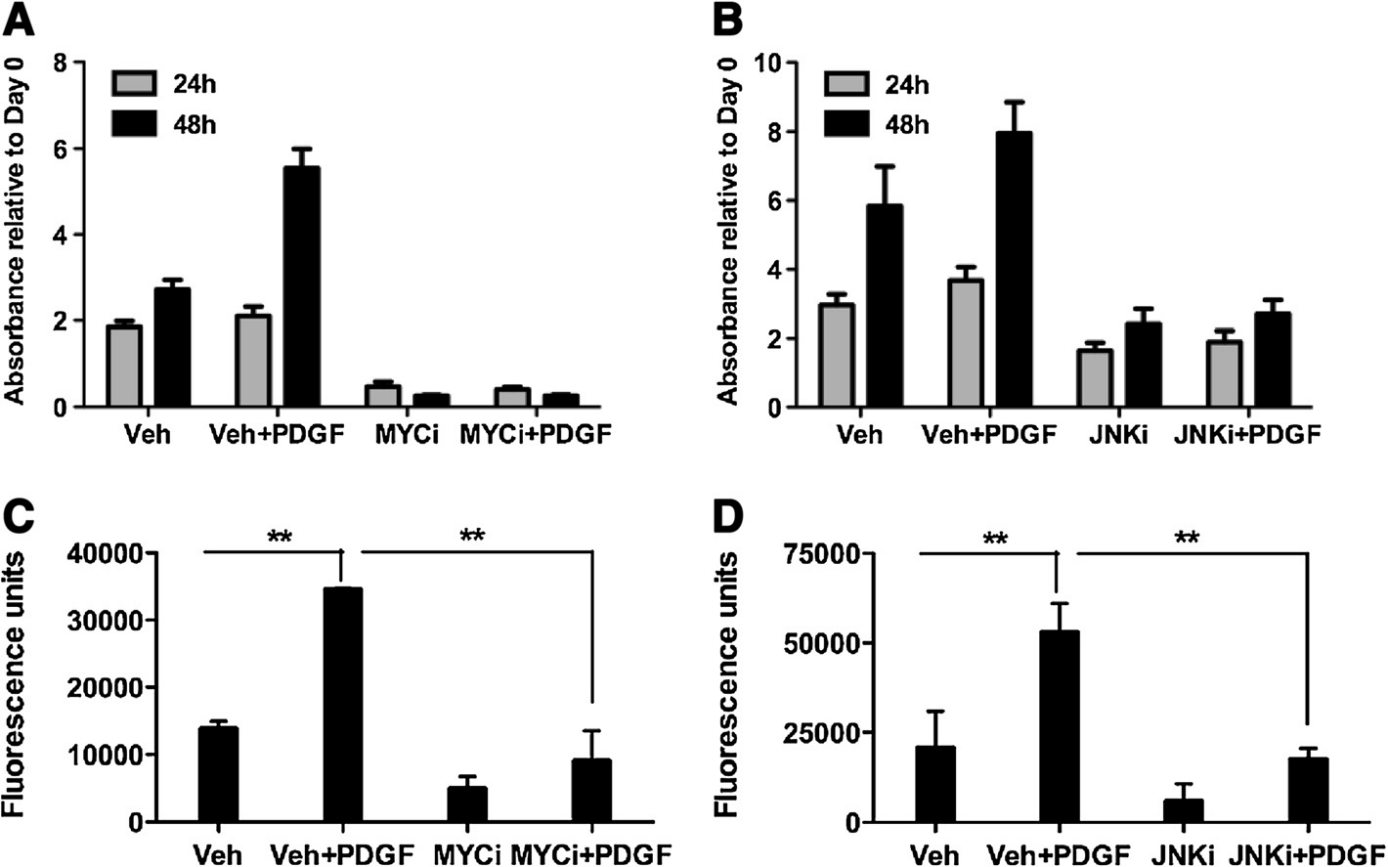
**Effect of inhibition of MYC and JNK/JUN function on PDGF-induced changes in cell growth and migration. (A, B)** Effects of MYC **(A)** and JUN **(B)** inhibition on the PDGF-stimulated increase in cell biomass, assessed 24 h or 48 h following treatment with growth factor **(C, D)** Inhibition of PDGF-directed cell migration in the presence of the MYC **(C)** and JUN **(D)** inhibitors. **, p < 0.05.

To determine the biological significance of MYC- and JUN-mediated transcriptional events, we measured the impact of pharmacologic inhibition of MYC and JUN activation on pBSMC proliferation and migration. Inhibition of MYC or JUN attenuated PDGF-induced pBSMC cell proliferation (Figures 6A and 6B) and migration (Figures 6C and 6D), respectively.

**Figure 7:**
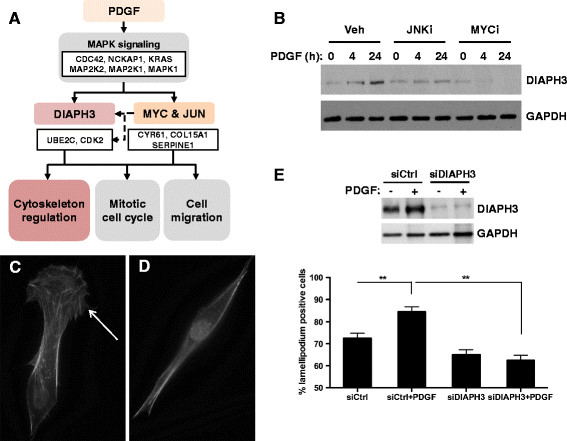
**DIAPH3 is a novel target of PDGF that regulates lamellipodium formation. (A)** Predictive model for MYC-JUN-DIAPH3 pathway in response to PDGF in pBSMC. **(B)** Immunoblot analysis depicting kinetics of DIAPH3 expression in pBSMC pre-treated with vehicle, JNK inhibitor (JNKi) or MYC inhibitor (MYCi) and subsequently treated with PDGF for 4 h or 24 h **(C, D)** Representative immunofluorescence images of pBSMC with **(C**, arrow**)** or without **(D)** lamellipodium formation. **(E)** Silencing of DIAPH3 attenuates the PDGF-mediated increase in lamellipodia formation in pBSMC (lower panel). Upper panel: immunoblot confirming efficiency of DIAPH3 knockdown in pBSMC. **, p < 0.05.

A common process underlying the dominant biological processes we identified (cell proliferation, chemotaxis, migration and angiogenesis) is actin cytoskeletal dynamics. Among the PDGF-responsive species identified at both the RNA and protein levels, the diaphanous-related formin protein DIAPH3 has been identified as a mediator of actin remodeling [30]–[32]. Our hypothetical model predicted a potential involvement of a MYC-JUN-DIAPH3 pathway in regulation of cytoskeletal remodeling in response to PDGF (Figure 7A). We investigated the effect of PDGF on DIAPH3 levels in pBSMC and demonstrated DIAPH3 down-regulation in PDGF-stimulated cells treated with MYC or JUN inhibitors (Figure 7B). RNAi-mediated silencing of DIAPH3 did not alter pBSMC proliferation or migration (data not shown), however it attenuated the PDGF-induced increase in lamellipodium formation in pBSMC (Figures 7C-E). Together, these findings suggest DIAPH3 may be a novel MYC and JUN target in pBSMC that regulates PDGF-induced alterations in cell morphology.

## Discussion

In this study we present a global analysis of gene and protein responses to PDGF in normal human visceral smooth muscle cells. To our knowledge this is the first integrated, quantitative proteomics and transcriptomics analysis in smooth muscle of any type. The proteomics dataset we have reported here represents the largest protein database of human SMCs ever assembled. Network analysis validated the importance of MYC and JUN/AP-1 in promoting SMC proliferation and migration, and also suggested the formin DIAPH3 may be a novel PDGF-sensitive regulator of SMC behavior. Our integrated analysis extends current understanding of PDGF-stimulated networks by uncovering a comprehensive list of PDGF-dependent biological processes and pathways and linking key transcription factors to their regulation. Moreover, integration of transcriptomics and proteomics revealed shared pathways, processes and master regulators. It also enhanced the reliability of both target identification and the associated network in comparison to microarray or proteomics analyses alone.

Pathologic remodeling of hollow organs such as the bladder, airways and vasculature involves alterations in SMC proliferation, extracellular matrix synthesis, cell morphology and cell motility. In agreement with these changes, integration analysis of differentially expressed genes and proteins in visceral SMC exposed to PDGF identified (i) regulation of cell proliferation; (ii) negative regulation of cell death; and (iii) regulation of cell motion as 3 of the most over-represented biological processes. A major finding of the current study was the emergence of MYC and JUN as dominant regulators of the PDGF-induced transcriptional program in visceral smooth muscle, and their identification as novel regulators of DIAPH3. Previous reports from us and others have implicated JUN/AP-1 in a variety of mechanosensitive cell behaviors in smooth muscle, including gene regulation, proliferation and migration [5],[24]–[26],[28],[33],[34]. Moreover, findings from our studies revealed significant overlap between mechanical and PDGF-stimulated signals in their ability to regulate signal transduction, gene expression and cell cycle transit [5],[26],[35]. In genome-wide expression profiling, we found that >70% of genes selectively induced by cyclic stretch-relaxation of SMC in vitro were similarly up-regulated by PDGF treatment [26]. In that study, informatics analysis revealed AP-1 as the transcription factor most significantly associated with stretch-induced gene expression. We proceeded to demonstrate that mechanical injury of the bladder promoted rapid phosphorylation of the PDGF receptor, independently of exogenous ligand, to promote up-regulation of the AP-1 target thrombomodulin [5]. Together, these observations suggest a mechanism underlying convergence of mechanical and growth factor signaling that involves PDGF receptor activation.

Among the overlapping genes and proteins identified in the current study as significantly enriched in response to PDGF treatment, CYR61, HMOX1 and CXCL12 emerged as genes linked to biological processes relevant to tissue remodeling, i.e. proliferation, migration and motility. Elevated CXCL12 and CYR61 have been implicated in fibroproliferative responses of vascular SMC and fibrocytes in arterial and airway remodeling [36]–[38], whereas CYR61 is elevated in hypertrophic smooth muscle of the bladder wall secondary to outlet obstruction and following cyclic stretch-relaxation of bladder SMC in vitro [27],[39]. Conversely, up-regulation of HMOX1 has been reported to attenuate both mitogen-induced proliferation and migration of SMC in vitro [40],[41], as well as smooth muscle remodeling in response to hypoxic injury [42]. In the current study, CYR61, HMOX1 and CXCL12 were also linked to the process of angiogenesis. A similar angiogenesis-focused gene signature was identified by Yang and colleagues in SMC exposed to mechanical stretch [27]. In that study AP-1, EGR-1 and MYB were identified as putative transcriptional regulators of the mechanosensitive transcriptional program, in agreement with our current and prior findings (Figure 2, [26]). Although MYC itself was not identified, the MYC family members upstream regulatory factor 1 (USF1) and USF2 were implicated as putative transcriptional regulators in both studies that evaluated stretch-induced gene expression in bladder SMC [27]. USF1 and USF2 bind to E-box motifs in target gene promoters and antagonize MYC activity [43],[44]. Notably, USF1 and USF2 have been shown to directly up-regulate transcription of HMOX-1 in vitro and in vivo [45],[46]. Our current findings showing that PDGF-induced downregulation of HMOX-1 in visceral SMC was reversed by pharmacologic inhibition of MYC is consistent with negative regulation of HMOX-1 expression by MYC and with its antagonistic interaction with USF1/2 at target gene regulatory regions. Exposure of hollow organs to mechanical stress in vivo induces transient hypoxia, as a result of vascular compression, which in turn enhances blood flow [47]. The identification of angiogenesis-associated gene signatures in SMC exposed to convergent mechanical or growth factor stimuli may therefore be a component of the subsequent hypertrophic and hyperplastic response that enables tissues to adapt to and counteract increased intraluminal pressure within the organ.

In a recent report, Yohannes and coworkers employed 2D-differential in-gel electrophoresis (2D-DIGE) to profile proteins that were differentially expressed in the bladder smooth muscle of rats subjected to streptozotocin-induced diabetes for different periods of time [48]. Diabetes promotes a spectrum of pathologic changes in the urinary tract, including profound alterations in smooth muscle mass and contractility [49]. Although not identified by 2D-DIGE as differentially expressed in experimental diabetes, MYC, along with EGR1 and the AP-1 subunit c-Fos, emerged as interconnected nodes following interrogation of differentially expressed proteins using MetaCore software [48]. Similarly, in our analysis, the transcription factors JUN, MYC and EGR1 were not identified as PDGF-induced proteins by quantitative proteomics analysis of primary SMC cultures, but were revealed through higher order transformation of expression data as master regulators of PDGF-stimulated transcriptional and protein changes in visceral SMC.

In the present study, analysis of the gene targets for each of the master regulators identified in Figure 2 revealed a high degree of potential cross-regulation, in that the promoter for each transcription factor contained putative binding sites for all other factors analyzed (data not shown). Consistent with the possibility for functional interaction, a recent study revealed time-dependent up-regulation of transcription factor-specific gene modules in an in vitro model of acute MYC activation [50]. In response to MYC induction, genes harboring AP-1 and CREB motifs were induced first, followed by those targeted by EGR1, and concluding with putative MYC targets. Taken together, these findings argue for a coordinated, temporal relationship between the master regulatory nodes we identified here. Given the potential for positive feedback regulation, they may also provide an explanation for the sustained fibroproliferation evident in hollow organ remodeling.

We further validated the network we have described by functional analysis of DIAPH3, which emerged as one of 22 targets that were induced at both mRNA and protein levels in response to PDGF. DIAPH3 is a member of the diaphanous-related formin family that regulates the actin and microtubule cytoskeletons downstream of the small Rho GTPases, Rho, Rac and Cdc42, in a variety of cell types (reviewed in [51]). Although primarily studied in epithelial cells and fibroblasts, the murine ortholog of DIAPH3, mDia2, has been implicated as a regulator of smooth muscle-specific gene expression in vascular SMC [52]. In that study, the primary activity of mDia2 and its homolog mDia1 was to enhance actin polymerization and thereby promote nuclear localization of the transcription factors MRTF-A and MRTF-B to induce expression of genes encoding smooth muscle contractile proteins. In the current study, silencing of DIAPH3 expression in visceral SMC did not affect migration or proliferation, but rather attenuated PDGF-stimulated formation of lamellipodia. These observations are consistent with a recent report describing a role for mDia2/DIAPH3 in nucleation of actin filaments in both filopodia and lamellipodia [32]. Notably, our prior quantitative proteomics study identified a cohort of actin cytoskeleton regulators that were up-regulated in caveolar lipid raft microdomains of PDGF-treated SMC [14]. Given the localization of activated PDGFR, actin regulators and DIAPH3 to lipid rafts ([14],[53],[54] and unpublished results, M.R.F), they support the functional importance of such microdomains as sites of integration for signals that regulate cell morphology and motility [55]–[57].

The mechanisms underlying regulation of DIAPH3 expression are largely unexplored. Our findings showed decreased expression of DIAPH3 in PDGF-treated SMC following pharmacologic inhibition of either JUN or MYC activity. Interestingly, the transcriptional co-activator Yes-associated protein (YAP) has been shown to promote DIAPH3 mRNA expression in fibroblasts [58] and to interact functionally with both JUN and MYC [59],[60]. Moreover, YAP is known to be upregulated in vascular SMC exposed to PDGF, and was found to be necessary for PDGF-mediated SMC proliferation [61]. Taken together, these findings are consistent with a direct role for MYC and/or JUN/AP-1 in transcription of the DIAPH3 gene.

## Conclusions

In summary, our results implicate MYC and JUN/AP-1 as key regulators of normal visceral SMC proliferation and migration, and provide the first evidence of a PDGF-sensitive MYC-regulated network in any cell type. These findings imply that MYC is a novel target for pharmacological intervention, not only in fibroproliferative expansion of smooth muscle in hollow organs, but also in cancers in which PDGFR-dependent signaling and/or MYC activation are drivers of tumor progression. Although transcription factors are challenging to target pharmacologically using small molecules, recent studies have reported encouraging results with inhibition of MYC in preclinical models of fibrosis and cancer [62]–[64]. Future studies evaluating these inhibitors in models of pathologic remodeling and cancer are clearly warranted.

## Materials and methods

### Materials

Recombinant human PDGF-BB was from R&D Systems (Minneapolis, MN). Antibodies to PDGFRα, PDGFRβ, phospho-PDGFRα/β Tyr849/Tyr857, c-Jun, phospho-c-Jun Ser63, c-Myc, EGR1, RUNX1, DDIT3, CYR61 and GDF15 were from Cell Signaling Technology (Danvers, MA); antibodies to Myb and NFAT5 were from Epitomics (Burlingame, CA); antibodies to SOX5 and GAPDH were from Santa Cruz Biotechnology (Santa Cruz, CA); antibody to β-actin was from Sigma Aldrich (Sigma Chemical Company, St. Louis, MO); antibody to DIAPH3 was a generous gift from Henry Higgs, Dartmouth Medical School. The c-Myc TF ELISA kit was from Active Motif (Carlsbad, CA). SP600125 and 10048-F4 were from EMD Biosciences (Billerica, MA). iScript cDNA synthesis reagents were from BioRad Laboratories (Hercules, CA). Universal PCR master mix for qRT-PCR and gene-specific assays were from Applied Biosystems (now Life Technologies, Grand Island, NY). Primers for human transcripts were as follows: Hs00171022_m1 for CXCL12; Hs00998500_g1 for CYR61; Hs01107330_m1 for DIAPH3; Hs02758991_g1 for GAPDH; Hs00171132_m1 for GDF15; Hs01110250_m1 for HMOX-1; Hs00998018_m1 for PDGFRA; and Hs01019589_m1 for PDGFRB. Primers for mouse transcripts were Mm00487499_g1 for Cyr61; Mm99999915_g1 for GAPDH; Mm00442228_m1 for Gdf15; Mm00435546_m1 for Pdgfrb.

### Cell culture and triplex SILAC labeling

Primary human bladder smooth muscle cells (pBSMCs) were cultured in smooth muscle cell medium (SMCM, Sciencell Research Laboratories, Carlsbad, CA) at 37°C in a humidified incubator with 5% CO_2_. For triplex SILAC labeling, pBSMCs were grown in arginine- and lysine-depleted SMCM (Sciencell Research Laboratories) supplemented with 2% (v/v) dialyzed fetal bovine serum (Invitrogen, Grand Island, NY) and L-arginine (Arg0) and L-lysine (Lys0), ^13^C_6_-L-arginine (Arg6) and 4,4,5,5-D_4_-L-lysine (Lys4), or ^13^C_6_^15^N_4_-L-arginine (Arg10) and ^13^C_6_^15^N_2_-L-lysine (Lys8) (Cambridge Isotope Laboratories, Andover, MA). After at least 6 population doublings, pBSMCs cultured in “light”, “medium”, and “heavy” SILAC media were serum starved overnight and treated with 1 nM PDGF-BB for 0, 4, and 24 h, respectively.

### RNA extraction and microarray analysis

After triplex SILAC labeling and PDGF treatment, RNAs were isolated from pBSMCs and hybridized to Human Gene 1.0 ST arrays (Affymetrix, Santa Clara, CA), which comprise 28,869 well-annotated genes. A quality assessment of the microarray data was performed essentially as described [65]. Several diagnostic plots including histogram and scatter plots of probe intensities in the arrays were used to check systemic bias of microarray experiments, such as high level of background intensity, signal saturation, and inter- and intra-group variation of the arrays. After the adjustment of background signal using the Plier method, probe intensities were normalized using the quantile normalization procedure with Affymetrix Expression Console software [66]. The raw data were deposited in the Gene Expression Omnibus (GSE52488).

### Identification of differentially expressed genes (DEGs)

With the normalized intensities, DEGs in samples at 4 h or 24 h after PDGF treatment in comparison with control samples were identified using an integrated statistical method previously described [50]. Briefly, two independent tests—the *T*-test and the log2 median ratio test—were performed. For each test, an empirical distribution of the null hypothesis that the means of the gene expression levels are not different was estimated by random permutations of the samples. For each gene, adjusted *p*-value was computed by performing a two-tailed test using the empirical distributions. The two sets of adjusted *p*-values were combined to compute the overall adjusted p-values using Stouffer’s method [67]. In addition, to determine the cutoff value of fold changes, we computed fold changes of randomly permuted samples and fitted a Gaussian distribution to the random fold changes. The 2.5 percentile (*i.e.,* the level of significance α = 0.05 in the two-tailed test) was calculated to be less than 1.4. Thus, the DEGs were selected based on the criteria that the overall *p* is less than 0.05 and that the absolute fold-change is larger than 1.4. Finally, to identify GOBPs or major pathways represented by the DEGs, the enrichment analysis was performed using the DAVID software [68]. Specifically enriched cellular processes between up- and down-regulation were selected with *p* < 0.05. Bar graphs were used to represent the level of significance of each cellular process with enrichment score (-log_10_*P*).

### Identification of key transcription factors (TFs) regulating DEGs

To identify key TFs, 278,346 TF-target interaction data points for 350 TFs were collected from public databases including TRED [17], EEDB [18], mSigDB [19], Amadeus [20], bZIPDB [21], and OregAnno [22]. The targets of each TF (TF_*i*_) were counted among the up- or down-regulated DEGs (*e.g., n* DEG targets of TF_*i*_). The same number of genes as up- or down-regulated DEGs were then randomly sampled from the whole genome and the target of TF_*i*_ in the randomly sampled genes was counted. This procedure was repeated 100,000 times. Next, an empirical distribution (null hypothesis distribution) of the 100,000 counts of random targets of TF_*i*_ was generated. For the number of targets of TF_*i*_, the probability (*P*) that the actual count of targets of TF_*i*_ in the DEGs can be observed by chance was computed using a one-tailed test with the empirical distribution. The *P* values of TF_*i*_ for up- and down-regulated DEGs were then combined using Stouffer’s method [67]. The same procedure was repeated for all TFs. Finally, eight TFs whose targets were significantly (combined *p* < 0.01) enriched by the DEGs were selected.

### Hierarchical clustering of DEGs and differentially expressed proteins (DEPs)

From the comparisons of 4 h versus 0 h and 24 h versus 0 h, we identified a total of 1,695 DEGs. We performed hierarchical clustering using Euclidean distance as the dissimilarity measure and the average linkage method: 4 clusters (Clusters 1-4) for DEGs that were up-regulated and 3 clusters (Clusters 5-7) for DEGs that were down-regulated (see heat maps in Figure 1B). The same clustering approach was applied in categorization of up- and down-regulated DEPs.

### Network model reconstruction

To reconstruct a sub-network describing regulatory target cellular processes by 5 key TFs in PDGF-perturbed pBSMCs, we first selected 255 target genes (from the 1,695 DEGs) of the 5 TFs, which are involved in 8 enriched cellular processes. We then built a network model describing the key TF-target interactions and protein-protein interactions among the targets. The TF-target interactions and protein-protein interactions of the 255 target genes and 5 key TFs were obtained from six databases: TRED [17], EEDB [18], mSigDB [19], Amadeus [20], bZIPDB [21], and OregAnno [22], for TF-target interactions, and HPRD [69], BioGRID [70], STRING [71] and KEGG [72] for protein-protein interactions. We downloaded all protein-protein interactions (PPIs) in HPRD, BioGRID, STRING, and KEGG and combined information from the four databases into one list. During this process, we converted protein IDs used in each database into Entrez IDs, converted directed PPIs from the KEGG pathway database into undirected PPIs, to be compatible with undirected PPIs obtained from the three databases, and generated a list of non-redundant interactions by removing redundant PPIs (i.e. multiple interactions) in the four databases. Also, by converting directed PPIs into undirected ones, the PPIs obtained from the databases should not be conflicting with each other. All these procedures were implemented in MATLAB. We then used Cytoscape version 2.8.2 to display PPIs. The nodes in the network with the same GOBPs [73] and KEGG pathway annotations [72] were arranged and grouped into the same network module. To quantitatively assess the regulatory potential of each key TF to 8 functional modules, we computed the fold enrichment score (FES) defined by (the number of target genes within a module)/(the total number of genes within the module)/(the total number of target genes in the network)/(the total number of genes in the network). This is a modified version of fold enrichment score from DAVID software [68].

### Protein preparation, separation, and tryptic digestion for mass spectrometric analysis

Whole cell lysates from differentially SILAC-labeled and PDGF-treated pBSMCs were extracted with RIPA lysis buffer. Protein concentrations were determined using Micro BCA assay (Thermo Fisher Scientific, Rockford, IL) according to the manufacturer’s protocol. Proteins extracted from SILAC-labeled pBSMCs were mixed in equal amounts. 40 μg of protein mixture was resolved on a 10% SDS-PAGE gel and visualized with Coomassie Blue R-250 staining solution. Each gel lane was excised into 10 slices of similar size and cut into approximately 1 mm^3^ particles prior to in-gel reduction, alkylation, and tryptic digestion as previously described [74]. Tryptic peptides were extracted, dried down in a SpeedVac (Thermo Savant, Holbrook, NY), and stored at -80°C until mass spectrometric analysis.

### Mass spectrometric analysis

Mass spectrometric analysis was conducted essentially as described [75]. Briefly, tryptic peptides were redissolved with 10 μL 1.5% acetic acid and 7.5% acetonitrile solution. 5 μL samples were analyzed by online C_18_ nanoflow reverse-phase HPLC (Eksigent nanoLC · 2D™, Dublin, CA) connected to an LTQ Orbitrap XL mass spectrometer (Thermo Fisher Scientific, Waltham, MA) essentially as described [76],[77]. Briefly, samples were loaded onto an in-house packed C_18_ column (Magic C_18_, 5 μm, 200Å) (Michrom Bioresources, Auburn, CA) with 15 cm length and 100 μm inner diameter, and separated at about 200 nl/min with 60 min linear gradients from 5 to 35% acetonitrile in 0.2% formic acid. Survey spectra were acquired in the Orbitrap analyzer with the resolution set to a value of 30,000. Lock mass option was enabled in all measurements and decamethylcyclopentasiloxane background ions (at m/z 371.10123) were used for real-time internal calibration. Up to five of the most intense ions per cycle were fragmented and analyzed in the linear ion trap.

### Protein identification and quantification

For protein identification and quantification, raw mass spectrometric data were analyzed with MaxQuant software (version 1.0.13.13) [78]. The parameters were set as follows. In the Quant module, SILAC triplets was selected; oxidation (M) and acetyl (Protein N-term) were set as variable modification; carbamidomethyl (C) was set as fixed modification; concatenated IPI human database (version 3.52) (74,190 forward sequences and 74,190 reverse sequences) was used for database searching; all other parameters were default. Tandem mass spectra were searched by Mascot (version 2.2.0.4) (Matrix Science, Boston, MA). In the Identify module, all parameters were default, except that maximal peptide posterior error probability was set as 0.05. False discovery rates for protein and peptide identifications were both set at 0.01.

### Identification of DEPs

Quality assessment of the SILAC datasets was performed as described [79]. The statistical analysis of the SILAC data and the calculation of fold-change cutoff were the same as for the microarray data. The DEPs were identified using the following criteria: 1) overall *P* values are less than 0.05; 2) proteins quantified in at least two replicates; and 3) absolute fold changes larger than 1.3.

### Assessment of correlation between PDGF perturbed transcriptome and proteome

Within each time point, correlation between normalized probe and SILAC intensity of genes and corresponding gene products product were estimated for the genes that had protein intensity data by Spearman’s rank correlation analysis. Relationships between fold change of DEGs and SILAC ratio of corresponding DEPs at 4 h and 24 h were estimated by the same method.

### Target validation by real-time RT-PCR

pBSMCs were seeded at a density of 100,000 cells per well in a 6-well plate, cultured for 24 h, serum starved for an additional 24 h, and then treated with 25 ng/ml (1 nM) PDGF-BB (R&D Systems, Minneapolis, MN) for the indicated times. After the treatment, cells were harvested in 500 μl Trizol reagent (Invitrogen, Carlsbad, CA). Total RNA was reverse transcribed using the iScript cDNA synthesis reagent (Bio-Rad, Hercules, CA) and cDNAs were amplified using gene-specific primers (Life Technologies, Grand Island, NY) according to the manufacturer’s instructions. In selected experiments cDNAs from a mouse model of bladder injury [28] were analyzed similarly. Briefly, injury was created in wild type female CD-1 mice, in which the proximal urethra was ligated with 6-0 nylon suture. Bladder distension injury was achieved by urine production by the mouse over a 24 h period. At the end of the experiment, tissues were harvested for analysis. Bladder smooth muscle was separated from the urothelium, prior to isolation of RNA and cDNA synthesis. All procedures were approved by the Institutional Animal Care and Use Committee. In each case relative abundance of each gene was normalized to levels of the housekeeping gene GAPDH. Quantification of gene expression was carried out using the 2^-ΔΔCt^ method.

### Immunoblot analysis

Immunoblot analysis was performed essentially as described [80]. Briefly, equal amounts of whole cell or tissue lysates were resolved by SDS-PAGE and electrotransferred to nitrocellulose membranes. Membranes were blocked with 10% non-fat dried milk in phosphate buffered saline containing 0.1% Tween-20 (PBS-T), rinsed with PBS-T, and incubated with protein-specific primary antibodies (1:1000 dilution) overnight at 4°C. After washing, membranes were incubated with species-specific HRP-conjugated secondary antibodies, and proteins were visualized following incubation with SuperSignal WestPico chemiluminescence reagent (Thermo Fisher Scientific, Rockford, IL) and exposure of membranes to X-ray film.

### Cell biomass and viability assays

Cell biomass was assessed using the crystal violet assay essentially as described [25]. Cells were fixed in 1% glutaraldehyde for 15 min and then in 0.5% (w/v) crystal violet solution for an additional 15 min. The plates were washed and dried overnight. 250 μl of Sorenson’s solution was added to each well and incubated for 15 min. Then the solution was transferred to a 96-well plate and the absorbance at 570 nm was measured using a FLUOstar Omega microplate reader (BMG LabTech, Durham, NC). To determine viability, cells were incubated in medium supplemented with 10% AlamarBlue reagent for 2 h at 37°C, 5% CO_2_. Relative fluorescence intensity of medium was measured as described [81].

### Transwell migration assays

After a 24 h serum depletion period, 1 × 10^6^ pBSMCs were nucleofected with 1 μg pmaxGFP (Amaxa, Inc., nucleofection program A033) and ~1.6 × 10^5^ cells seeded in each of four transwell FluoroBlok™ inserts (BD Biosciences, San Jose, CA) containing 500 μL serum-free SMCM with JNK inhibitor, MYC inhibitor or vehicle (DMSO). The transwells were placed in the corresponding wells of a companion plate containing 1 ml/well serum-free SMCM. 25 ng/ml PDGF-BB was added 60 min later to the SMCM in the bottom wells. The remaining cells were seeded in two wells of a six-well plate for confirmation of transfection efficiency. At the indicated times after adding PDGF, transwell inserts were rinsed three times with PBS for 5 min and then transferred to a glass-bottomed 24-well black plate (Greiner, Monroe, NC). GFP fluorescence signal was measured with a FLUOstar Omega microplate reader (BMG LabTech) using the bottom optic, with excitation and emission wavelengths of 485 nm and 520 nm, respectively.

### DIAPH3 functional assay

1 × 10^6^ pBSMCs were nucleofected as described above with 1 μg pmaxGFP and 1 μM DIAPH3 siRNA or non-targeting control. 10,000 cells from each nucleofection mix were seeded onto sterile coverslips in 6-well plates for 24 h. Following a 24 h serum depletion, cells were treated without or with 1 nM PDGF-BB and harvested after 24 h for assessment of lamellipodia formation. Briefly, cells were fixed for 10 min in 4% paraformaldehyde with gentle shaking, followed by 2 washes for 5 min each with PBS. Cells were permeabilized with 0.1% Triton X-100 in PBS for 5-10 min, washed and incubated in blocking buffer (PBS containing 1% goat serum and 0.2% BSA) for an hour, with gentle shaking. Cells were washed 3 times with 0.2% BSA/PBS for 5 min each and incubated in a 1:1000 solution of rhodamine-phalloidin (Invitrogen, Grand Island, NY) in 0.2% BSA/PBS for 1 h with gentle shaking. Finally, cells were washed 3 times with PBS for 5 min each and the coverslips mounted onto slides in Vectashield mounting medium containing DAPI. The slides were allowed to dry overnight at 4°C prior to imaging on a Zeiss Axioplan 2 microscope. Cells were scored as lamellipodia-positive or negative by two independent observers (A.R and S.M.), from three independent trials, using at least 50 cells per condition, and data combined for determination of statistical significance.

### Statistical analysis

In most cases, comparisons between experimental groups were performed using Student’s t-test. P values are indicated in figure legends. Real-time RT-PCR data between conditions were analyzed using the non-parametric Mann-Whitney test. For comparison of lamellipodia formation data were analyzed using a linear model with fixed conditions (siDIAPH3/siControl, PDGF+/Vehicle, Rater, Experimental Run) and interaction terms between PDGF and condition, and Experimental Run and Rater were fit to the ratio of lamellipodium-positive cells to total number of cells. The diagnostic plots were examined. No transformations were necessary to the outcome variable, and no violations of model assumptions were observed on the plots. The Tukey Honestly Significant difference test was used to determine statistical significance of the difference in cell ratios between each pair of conditions. Statistical analyses were performed using R statistical software packages base (http://www.R-project.org/) and multcomp [82].

### Supporting data

The datasets supporting the results of this article are available in the Gene Expression Omnibus (GSE52488, http://www.ncbi.nlm.nih.gov/geo/query/acc.cgi?acc=GSE52488) and in the ProteomeXchange Consortium (http://proteomecentral.proteomexchange.org) via the PRIDE partner repository [83], with the dataset identifier PXD000624 and doi:10.6019/PXD000624).

## Abbreviations

AP-1: Activator Protein 1

bioGRID: Biological General Repository for Interaction Datasets

BSA: Bovine Serum Albumin

bZIPDB: Basic region-leucine zipper transcription factor database

CXCL12: Chemokine (C-X-C motif) ligand 12, also known as stromal cell-derived factor 1

CYR61: Cysteine-rich, angiogenic inducer, 61

DAPI: 4',6-diamidino-2-phenylindole

DAVID: Database for Annotation, Visualization and Integrated Discovery

DEG: Differentially Expressed Gene

DEP: Differentially Expressed Protein

DIAPH3: Diaphanous-related formin 3

DMSO: Dimethylsulfoxide

EEDB: EdgeExpress database

FDR: False Discovery Rate

FES: Fold Enrichment Score

GAPDH: Glyceraldehyde-3-phosphate dehydrogenase

GDF15: Growth and Differentiation Factor 15

GFP: Green Fluorescent Protein

GOBP: Gene Ontology biological processes

HMOX-1: Heme Oxygenase-1

HPLC: High Performance Liquid Chromatography

HPRD: Human Protein Reference Database

JNKi: JNK inhibitor, SP600125

KEGG: Kyoto Encyclopedia of Genes and Genomes

mSigDB: Molecular signatures database

MRFT-A/B: Myocardin-related transcription factor-A/B

MYCi: MYC inhibitor, 10058-F4

NFAT: Nuclear Factor of Activated T-cells

pBSMC: Primary bladder smooth muscle cell

PDGF-BB: Platelet-derived growth factor, BB isoform

PDGFR: Platelet-derived growth factor receptor

PPI: Protein-protein interaction

RT-PCR: Reverse transcription polymerase chain reaction

SDS-PAGE: Sodium dodecyl sulfate polyacrylamide gel electrophoresis

SILAC: Stable Isotope Labeling of Amino acids in Cell Culture

SMC: Smooth muscle cell

STRING: Search Tool for the Retrieval of Interacting Genes/proteins

TF: Transcription factor

TRED: Transcriptional Regulatory Element Database

USF1/2: Upstream regulatory factor 1/2

## Competing interests

The authors declare that they have no competing interests.

## Authors’ contributions

WY performed mass spectrometry and analysis of proteomics data; AR conducted validation assays and data analysis; SY performed integration analysis on transcriptomic and proteomic datasets as well as network modeling and construction; HJ assisted with transformation and analysis of transcriptomic and proteomic datasets; SM performed in vitro assays; MDM assisted with validation assays; TL performed statistical analysis; JK participated in integration and network analysis; DH supervised integration analysis, network modeling and construction and assisted with interpretation. MRF conceived of the study and wrote the manuscript; RMA conceived of the study, performed validation assays and wrote the manuscript. All authors read and approved the final manuscript.

## Additional files

## Supplementary Material

Additional file 1: Figure S1. PDGFR expression in vitro and in vivo. Primary human bladder smooth muscle cells (pBSMC) were analyzed for relative expression of PDGFRA and PDGFRB isoforms by real-time RT-PCR **(A)** or immunoblot analysis of 5 or 10 μg pBSMC lysate with the indicated antibodies **(B)**. **(C)** cDNAs from bladder muscle from the indicated strains, genders and ages of mice were analyzed for relative expression of PDGFRα and PDGFRβ isoforms by real-time RTPCR. **(D)** Cell lysates from pBSMC treated with PDGF for the indicated times (in h) were subjected to immunoblot analysis using the specified antibodies. Data are representative of at least 2 trials. Click here for file

Additional file 2: Figure S2. Workflow for the quantitative transcriptomics and proteomics analyses of pBSMCs in response to PDGF treatment. pBSMCs were triplex SILAC labeled and treated with PDGF for 0, 4, and 24 h. RNAs were isolated from each population of pBSMCs and analyzed on Human Gene 1.0 ST arrays. Proteins were extracted from each population of pBSMCs and mixed at a 1:1:1 (w/w/w) ratio. The protein mixture was analyzed by gel-enhanced liquid chromatography-tandem mass spectrometry (GeLC-MS/MS). The transcriptomics and proteomics datasets were analyzed to construct a putative network model for the molecules regulated by PDGF in pBSMCs. Click here for file

Additional file 3: Figure S3. Quality assessment of microarray data. **(A)** The histogram shows density of the microarray data. As shown in the figure there are no significant differences between the distribution of 12 samples in terms of shape and range after normalization with quantile method, demonstrating no problems with high level of background intensity and signal saturation. **(B)** The scatter plots illustrate reproducibility based on inter- and intra-group variations of the arrays. The diagonal shows the intensity distribution in each array. All pairwise correlation coefficients between samples were > 0.98. The Pearson correlation coefficient within groups was higher (>0.995) than those between groups. Click here for file

Additional file 4: Table S1. A total 1,695 DEGs perturbed by PDGF stimulation. Click here for file

Additional file 5: Figure S4. Representative mass spectra for triplex SILAC quantification. **(A)** and **(B)** show a trio of SILAC peptides derived from hippocalcin-like protein 1 (HPCAL1), which was significantly upregulated by PDGF treatment in two-dimensional (2D) and three-dimensional (3D) modes, respective. **(C)** and **(D)** show a trio of SILAC peptides derived from β-type PDGF receptor (PDGFRB), which was significantly downregulated by PDGF treatment in 2D and 3D modes, respective. In the MaxQuant-generated 3D pictures, the SILAC peptide trios were shown as 3D objects in *m/z*, elution time, and signal intensity space. Click here for file

Additional file 6: Table S2. 241 DEPs perturbed in response to PDGF treatment. Click here for file

Additional file 7: Figure S5. Overall correlation between the proteome and transcriptome. Relationship between PDGF perturbed protein and gene expression. Correlations between SILAC intensities and normalized probe intensities at each time point were estimated by Spearman’s rank correlation analysis to determine the correlation between all identified genes by microarray analysis and proteins by SILAC-based proteomics analysis. Click here for file

Additional file 8: Table S3. 40 common mRNA and protein species in response to PDGF treatment. Click here for file

Additional file 9: Figure S6. Confirmation of JNK and MYC inhibitor efficacy. **(A)** Nuclear extracts prepared from pBSMC treated with PDGF for various time periods were assessed for DNA binding activity of MYC using a transcription factor ELISA. Nuclear extract from Jurkat cells was included as a positive control. **(B)** TF ELISA depicting a reduction in DNA-binding function of MYC in nuclear extracts prepared from pBSMC pre-treated for an hour with 32 μM MYC inhibitor followed by stimulation with PDGF for 2 hours. **(C)** Immunoblot confirming efficacy of the JNK inhibitor as evidenced by lack of c-Jun phosphorylation in pBSMC pre-treated for an hour with the inhibitor, followed by exposure to PDGF for 4 h or 24 h. Click here for file
