# Supplementary figures and images for: Integration of proteomic and transcriptomic profiles identifies a novel PDGF-MYC network in human smooth muscle cells
Source: Cell Commun Signal. 2014 Aug 1;12:44. doi: 10.1186/s12964-014-0044-z (PMC4422302; doi:10.1186/s12964-014-0044-z)

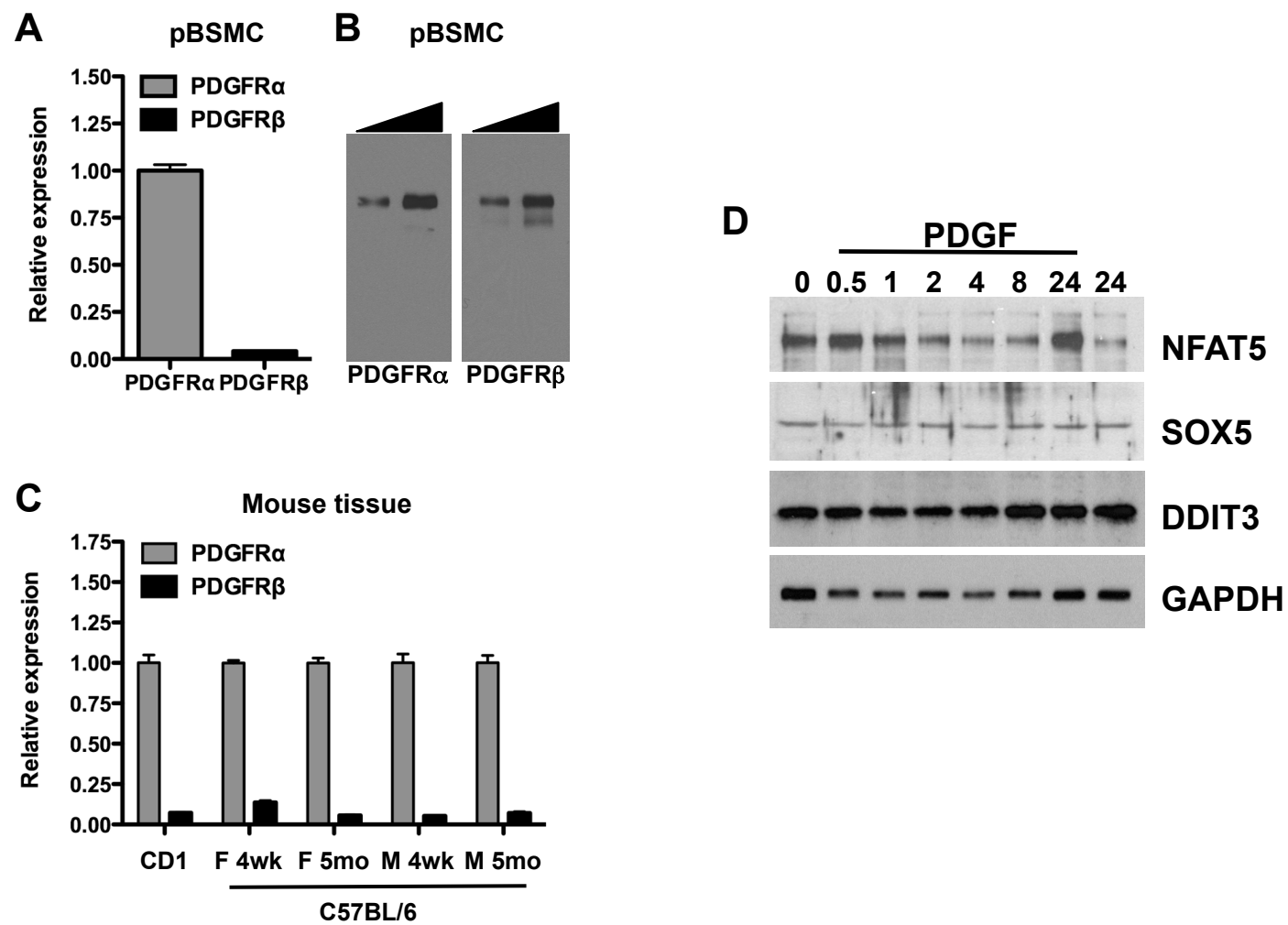

Figure S2

Supplement: Additional file 1: Figure S1. — PDGFR expression in vitro and in vivo. Primary human bladder smooth muscle cells (pBSMC) were analyzed for relative expression of PDGFRA and PDGFRB isoforms by real-time RT-PCR (A) or immunoblot analysis of 5 or 10 μg pBSMC lysate with the indicated antibodies (B). (C) cDNAs from bladder muscle from the indicated strains, genders and ages of mice were analyzed for relative expression of PDGFRα and PDGFRβ isoforms by real-time RTPCR. (D) Cell lysates from pBSMC treated with PDGF for the indicated times (in h) were subjected to immunoblot analysis using the specified antibodies. Data are representative of at least 2 trials. [file s12964-014-0044-z-S1.pdf]

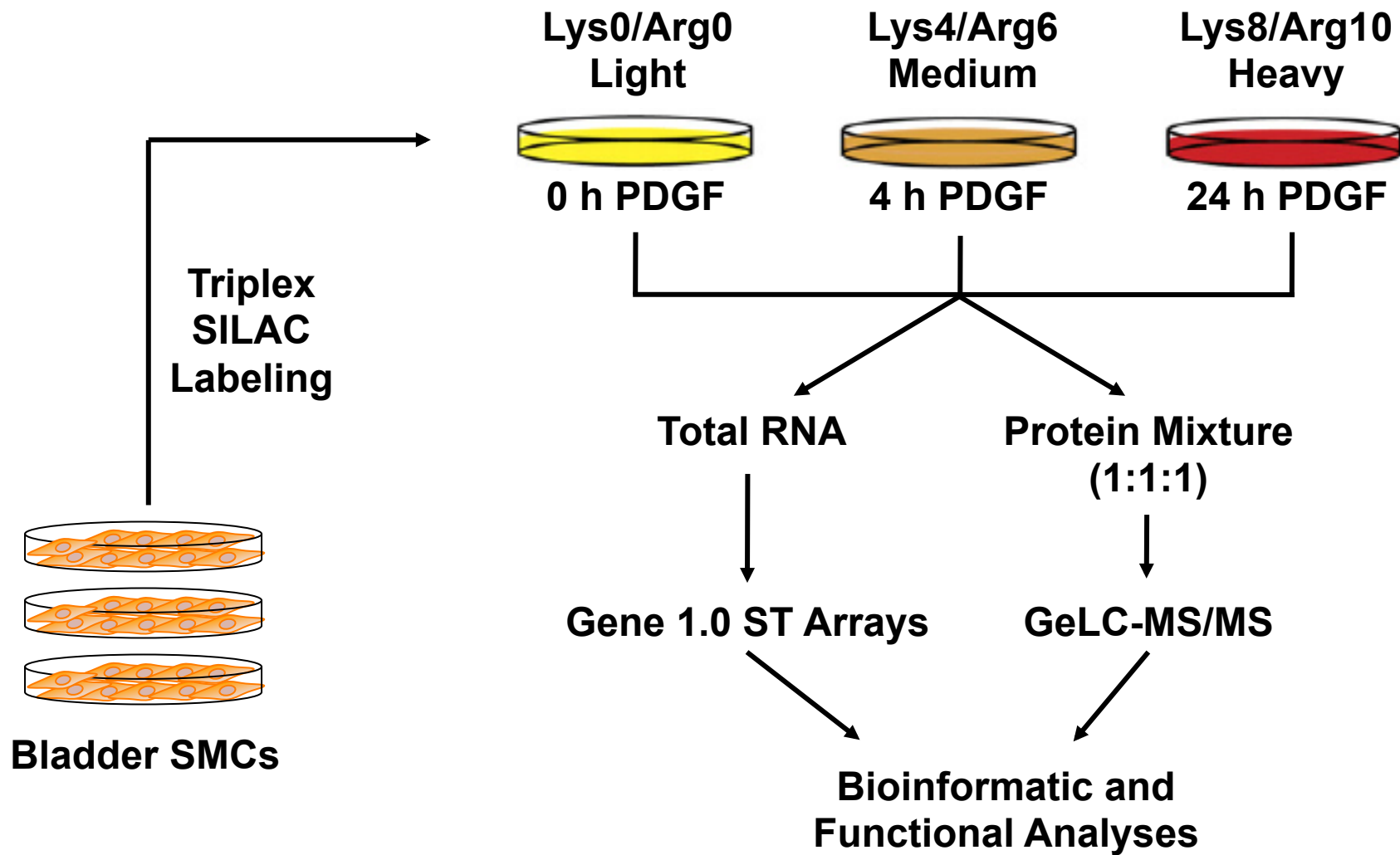

Figure S1

Supplement: Additional file 2: Figure S2. — Workflow for the quantitative transcriptomics and proteomics analyses of pBSMCs in response to PDGF treatment. pBSMCs were triplex SILAC labeled and treated with PDGF for 0, 4, and 24 h. RNAs were isolated from each population of pBSMCs and analyzed on Human Gene 1.0 ST arrays. Proteins were extracted from each population of pBSMCs and mixed at a 1:1:1 (w/w/w) ratio. The protein mixture was analyzed by gel-enhanced liquid chromatography-tandem mass spectrometry (GeLC-MS/MS). The transcriptomics and proteomics datasets were analyzed to construct a putative network model for the molecules regulated by PDGF in pBSMCs. [file s12964-014-0044-z-S2.pdf]

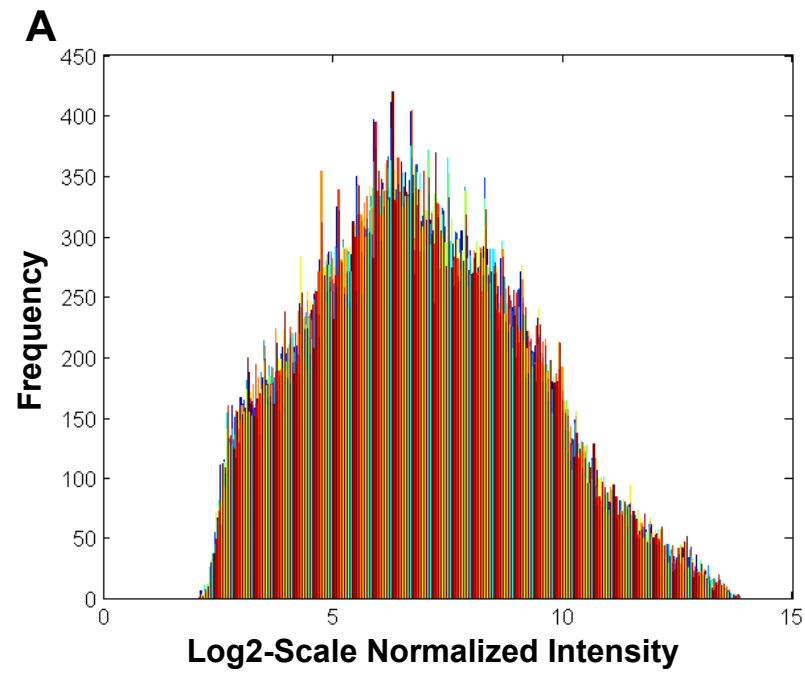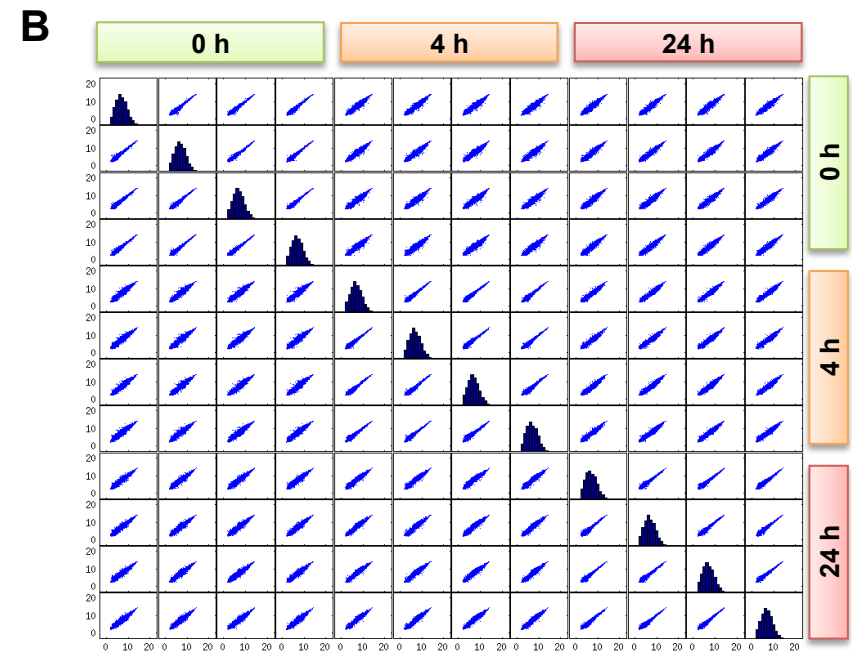

Figure S3

Supplement: Additional file 3: Figure S3. — Quality assessment of microarray data. (A) The histogram shows density of the microarray data. As shown in the figure there are no significant differences between the distribution of 12 samples in terms of shape and range after normalization with quantile method, demonstrating no problems with high level of background intensity and signal saturation. (B) The scatter plots illustrate reproducibility based on inter- and intra-group variations of the arrays. The diagonal shows the intensity distribution in each array. All pairwise correlation coefficients between samples were > 0.98. The Pearson correlation coefficient within groups was higher (>0.995) than those between groups. [file s12964-014-0044-z-S3.pdf]

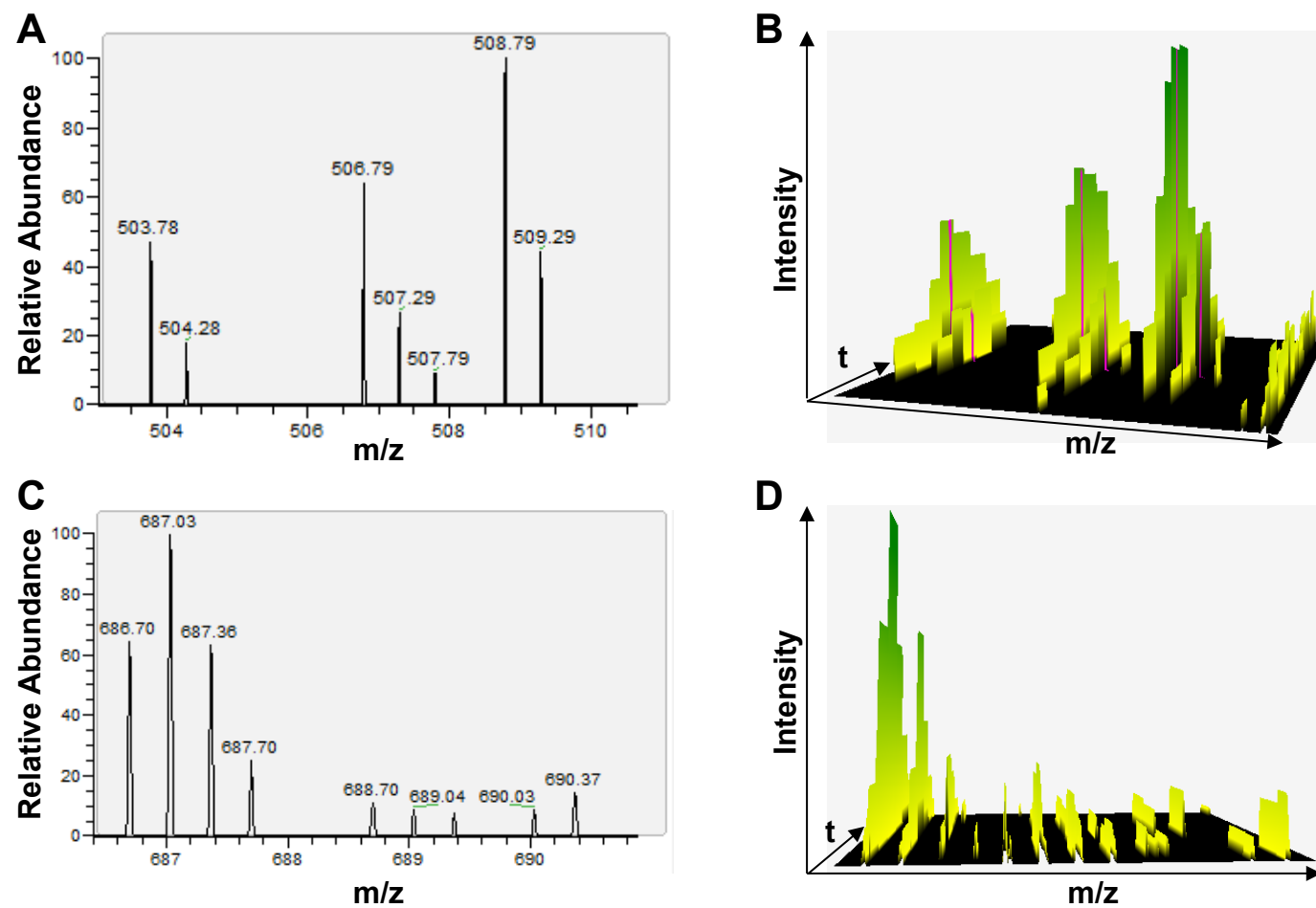

Figure S4

Supplement: Additional file 5: Figure S4. — Representative mass spectra for triplex SILAC quantification. (A) and (B) show a trio of SILAC peptides derived from hippocalcin-like protein 1 (HPCAL1), which was significantly upregulated by PDGF treatment in two-dimensional (2D) and three-dimensional (3D) modes, respective. (C) and (D) show a trio of SILAC peptides derived from β-type PDGF receptor (PDGFRB), which was significantly downregulated by PDGF treatment in 2D and 3D modes, respective. In the MaxQuant-generated 3D pictures, the SILAC peptide trios were shown as 3D objects in m/z, elution time, and signal intensity space. [file s12964-014-0044-z-S5.pdf]

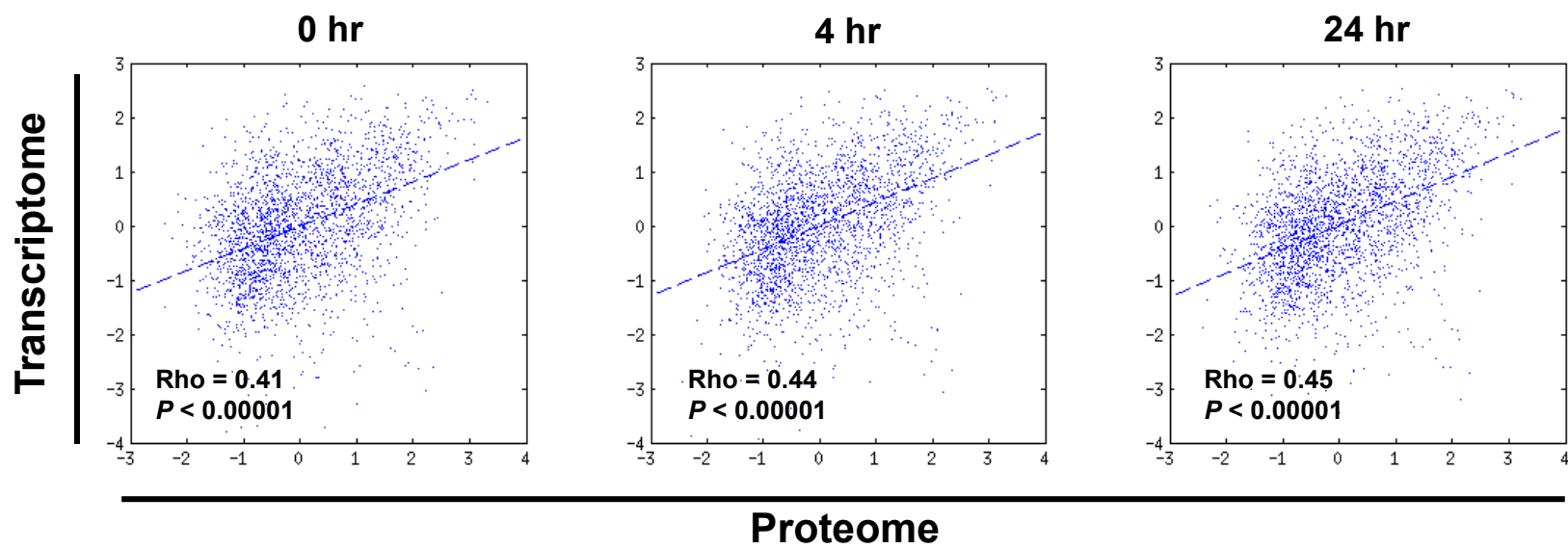

Figure S5

Supplement: Additional file 7: Figure S5. — Overall correlation between the proteome and transcriptome. Relationship between PDGF perturbed protein and gene expression. Correlations between SILAC intensities and normalized probe intensities at each time point were estimated by Spearman’s rank correlation analysis to determine the correlation between all identified genes by microarray analysis and proteins by SILAC-based proteomics analysis. [file s12964-014-0044-z-S7.pdf]

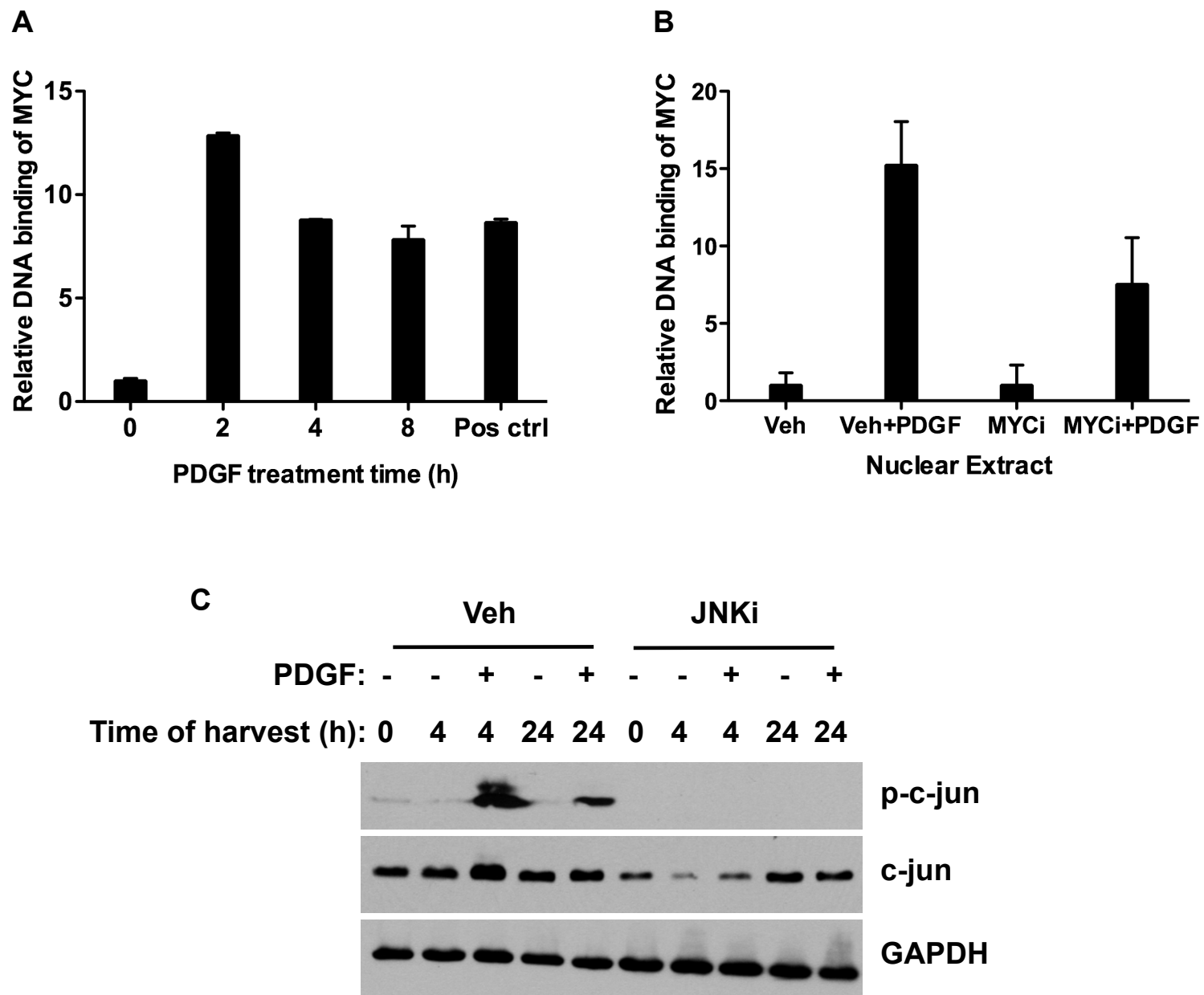

Figure S6

Supplement: Additional file 9: Figure S6. — Confirmation of JNK and MYC inhibitor efficacy. (A) Nuclear extracts prepared from pBSMC treated with PDGF for various time periods were assessed for DNA binding activity of MYC using a transcription factor ELISA. Nuclear extract from Jurkat cells was included as a positive control. (B) TF ELISA depicting a reduction in DNA-binding function of MYC in nuclear extracts prepared from pBSMC pre-treated for an hour with 32 μM MYC inhibitor followed by stimulation with PDGF for 2 hours. (C) Immunoblot confirming efficacy of the JNK inhibitor as evidenced by lack of c-Jun phosphorylation in pBSMC pre-treated for an hour with the inhibitor, followed by exposure to PDGF for 4 h or 24 h. [file s12964-014-0044-z-S9.pdf]
